# *Elaphroporia ailaoshanensis* gen. et sp. nov. in Polyporales (Basidiomycota)

**DOI:** 10.3897/mycokeys.29.22086

**Published:** 2018-01-30

**Authors:** Zi-Qiang Wu, Tai-Min Xu, Shan Shen, Xiang-Fu Liu, Kai-Yue Luo, Chang-Lin Zhao

**Affiliations:** 1 Key Laboratory of Forest Disaster Warning and Control of Yunnan Province, Southwest Forestry University, Kunming 650224, P.R. China; 2 College of Life Sciences, Southwest Forestry University, Kunming 650224, P.R. China; 3 College of Biodiversity Conservation and Utilisation, Southwest Forestry University, Kunming 650224, P.R. China

**Keywords:** Meruliaceae, phylogeny, polypore, taxonomy, wood-inhabiting fungi

## Abstract

A new poroid wood-inhabiting fungal genus, *Elaphroporia*, typified by *E. ailaoshanensis* **sp. nov.**, is proposed based on a combination of morphological features and molecular evidence. The genus is characterised by an annual growth habit, resupinate basidiocarps, becoming rigid and light-weight up on drying, a monomitic hyphal system with thick-walled generative hyphae bearing both clamp connections and simple septa, slightly amyloid, CB+ and ellipsoid, hyaline, thin-walled, smooth and IKI–, CB– basidiospores. Sequences of ITS and LSU nrRNA gene regions of the studied samples were generated, and phylogenetic analyses were performed with maximum likelihood, maximum parsimony and bayesian inference methods. The phylogenetic analysis based on molecular data of ITS+nLSU sequences showed that *Elaphroporia* belonged to the residual polyporoid clade and was closely related to *Junghuhnia crustacea*. Further investigation was obtained for more representative taxa in the Meruliaceae based on ITS+nLSU sequences, in which the result demonstrated that the genus *Elaphroporia* formed a monophyletic lineage with a strong support (100 % BS, 100 % BP, 1.00 BPP) and then grouped with *Flaviporus* and *Steccherinum*.

## Introduction

The Polyporales is a large group of Agaricomycetes and includes more than 1800 taxa at species level belonging to 216 genera and 13 families (Kirk et al. 2008). Species in Polyporales are the key players amongst the wood-rotting fungi because of their importance in the carbon cycle (Floudas et al. 2012) and the pathogenic and potential application in biomedical engineering and biodegradation (Dai et al. 2009, Levin et al. 2016).

Molecular systematics has played a powerful role in inferring phylogenies within fungal groups since the early 1990s (White et al. 1990, Hibbett et al. 2007, Larsson 2007, Miettinen et al. 2011, Binder et al. 2013, Dai et al. 2015, Choi and Kim 2017). Recently, molecular studies involving Meruliaceae P. Karst. have been carried out (Binder et al. 2005, 2013, Miettinen and Larsson 2011, Miettinen and Rajchenberg 2012, Hibbett et al. 2016, Miettinen et al. 2016).

Larsson (2007) introduced a new division for part of the Polyporales, effectively renaming the phlebioid and residual polyporoid clades as the Meruliaceae, Phanerochaetaceae Jülich, and *Byssomerulius* Parmasto families. A phylogenetic study of Meruliaceae employing multi-genes suggested that 1) this family included species with both poroid and hydnoid hymenophore configurations, and 2) the genera of *Flabellophora* G. Cunn., *Flaviporus* Murrill, *Junghuhnia* Corda, *Steccherinum* Gray and *Xanthoporus* Audet belong to this family (Miettinen et al. 2011). Moreover, further study employing a six-gene (5.8S, nrLSU, nrSSU, rpb1, rpb2, tef1) dataset has constructed a phylogenetic and phylogenomic overview of the Polyporales, which showed that the species of Meruliaceae fall into the residual polyporoid clade (Binder et al. 2013).

Wood-rotting fungi is a cosmopolitan group and it has a rich diversity on the basis of growing on boreal, temperate, subtropical, and tropical vegetations (Gilbertson and Ryvarden 1987, Núñez and Ryvarden 2001, Dai 2012, Ryvarden and Melo 2014, Dai et al. 2015). During investigations on wood-inhabiting fungi in southern China, an additional taxon was found which could not be assigned to any described genus. It produces annual, resupinate basidiocarps, a monomitic hyphal system with generative hyphae bearing both simple septa and clamp connections, slightly amyloid, CB+ and ellipsoid, hyaline, thin-walled, smooth basidiospores. These characters make it distinguishable from all known poroid and hydnoid wood-inhabiting fungal genera (Gilbertson and Ryvarden 1987, Núñez and Ryvarden 2001, Bernicchia and Gorjón 2010, Ryvarden and Melo 2014). In this study, the authors expand samplings from previous studies to examine taxonomy and phylogeny of this new genus within the Polyporales, based on the internal transcribed spacer (ITS) regions and the large subunit nuclear ribosomal RNA gene (nLSU) sequences.

## Materials and methods

*Morphological studies*. The specimens studied are deposited at the herbarium of Southwest Forestry University (SWFC). Macro-morphological descriptions are based on field notes. Special colour terms follow Petersen (1996). Micro-morphological data were obtained from the dried specimens and observed under a light microscope following Dai (2010). The following abbreviations were used: KOH = 5% potassium hydroxide, CB = Cotton Blue, CB– = acyanophilous, IKI = Melzer’s reagent, IKI– = both inamyloid and indextrinoid, IKI+ = amyloid, L = mean spore length (arithmetic average of all spores), W = mean spore width (arithmetic average of all spores), Q = variation in the L/W ratios between the specimens studied, n (a/b) = number of spores (a) measured from given number (b) of specimens.

*DNA extraction and sequencing*. CTAB rapid plant genome extraction kit-DN14 (Aidlab Biotechnologies Co., Ltd, Beijing) was used to obtain genomic DNA from dried specimens, according to the manufacturer’s instructions with the modification that a small piece of dried fungal specimen (about 30 mg) was ground to powder with liquid nitrogen. The powder was transferred to a 1.5 ml centrifuge tube, suspended in 0.4 ml of lysis buffer and incubated in a 65 °C water bath for 60 min. After that, 0.4 ml phenol-chloroform (24:1) was added to each tube and the suspension was shaken vigorously. After centrifugation at 13 000 rpm for 5 min, 0.3 ml supernatant was transferred to a new tube and mixed with 0.45 ml binding buffer. The mixture was then transferred to an adsorbing column (AC) for centrifugation at 13 000 rpm for 0.5 min. Then, 0.5 ml inhibitor removal fluid was added in AC for a centrifugation at 12 000 rpm for 0.5 min. After washing twice with 0.5 ml washing buffer, the AC was transferred to a clean centrifuge tube, and 100 ml elution buffer was added to the middle of the adsorbed film to elute the genome DNA. The ITS region was amplified with primer pairs ITS5 and ITS4 (White et al. 1990). The nuclear LSU region was amplified with primer pairs LR0R and LR7 (https://sites.duke.edu/vilgalyslab/rdna_primers_for_fungi/). The PCR procedure for ITS was as follows: initial denaturation at 95 °C for 3 min, followed by 35 cycles at 94 °C for 40 s, 58 °C for 45 s and 72 °C for 1 min, and a final extension of 72 °C for 10 min. The PCR procedure for nLSU was as follows: initial denaturation at 94 °C for 1 min, followed by 35 cycles at 94 °C for 30 s, 48 °C for 1 min and 72 °C for 1.5 min, and a final extension of 72 °C for 10 min. The PCR products were purified and directly sequenced at Kunming Tsingke Biological Technology Limited Company. All newly generated sequences were deposited at GenBank (Table 1).

**Table 1.** A list of species, specimens and GenBank accession number of sequences used in this study.

Species name	Sample no.	GenBank accession no.		References
		ITS	nLSU	
*Abortiporus biennis*	TFRI 274	EU232187	EU232235	Larsson (2007)
*Antrodia albida*	CBS 308.82	DQ491414	AY515348	Kim et al. (2007)
*Antrodia heteromorpha*	CBS 200.91	DQ491415	AY515350	Kim et al. (2007)
*Antrodiella americana*	Gothenburg 3161	JN710509	JN710509	Miettinen et al. (2011)
*Antrodiella pallasii*	Renvall 89a	AF126896	–	Binder et al. (2013)
*Antrodiella semisupina*	FCUG 960	EU232182	EU232266	Binder et al. (2005)
*Antrodiella* sp.	X 418	JN710523	JN710523	Miettinen et al. (2011)
*Atraporiella neotropica*	Ryvarden 44447	HQ659221	HQ659221	Miettinen and Rajchenberg (2012)
*Ceriporia viridans*	Dai 7759	KC182777	–	Jia et al. (2014)
*Ceriporiopsis balaenae*	H7002389	FJ496669	FJ496717	Tomšovský et al. (2010)
*Ceriporiopsis consobrina*	Rivoire 977	FJ496667	FJ496716	Tomšovský et al. (2010)
*Ceriporiopsis gilvesce*ns	BRNM 667882	FJ496685	FJ496719	Tomšovský et al. (2010)
*Ceriporiopsis gilvescens*	BRNM 710166	FJ496684	FJ496720	Tomšovský et al. (2010)
*Ceriporiopsis gilvescens*	Yuan 2752	KF845946	KF845953	Zhao and Cui (2014)
*Ceriporiopsis guidella*	HUBO 7659	FJ496687	FJ496722	Tomšovský et al. (2010)
*Cinereomyces lindbladii*	FBCC 177	HQ659223	HQ659223	Miettinen and Rajchenberg (2012)
*Climacocystis borealis*	KH 13318	JQ031126	JQ031126	Binder et al. (2013)
*Coriolopsis caperata*	LE(BIN)-0677	AB158316	AB158316	Tomšovský et al. (2010)
*Dacryobolus karstenii*	KHL 11162	EU118624	EU118624	Binder et al. (2005)
*Daedalea quercina*	DSM 4953	DQ491425	DQ491425	Kim et al. (2007)
*Diplomitoporus flavescens*	X 84	FN907908	–	Miettinen et al. (2011)
*Earliella scabrosa*	PR1209	JN165009	JN164793	Justo and Hibbett (2011)
*Etheirodon fimbriatum*	Larsson 11905	JN710530	JN710530	Miettinen et al. (2011)
*Flabellophora* sp.1	X 1357	JN710533	JN710533	Miettinen et al. (2011)
*Flabellophora* sp.2	X 340	JN710534	JN710534	Miettinen et al. (2011)
*Flabellophora* sp.3	X 1277	JN710535	JN710535	Miettinen et al. (2011)
*Flabellophora* sp.4	X 439	JN710536	JN710536	Miettinen et al. (2011)
*Flaviporus brownii*	X 1216	JN710537	JN710537	Miettinen et al. (2011)
*Flaviporus liebmannii*	X 251	JN710541	JN710541	Miettinen et al. (2011)
*Flaviporus liebmannii*	X 249	JN710539	JN710539	Miettinen et al. (2011)
*Flaviporus liebmannii*	X 666	JN710540	JN710540	Miettinen et al. (2011)
*Fomitopsis pinicola*	CBS 221.39	DQ491405	DQ491405	Kim et al. (2007)
*Fomitopsis rosea*	ATCC 76767	DQ491410	DQ491410	Kim et al. (2007)
*Fragiliporia fragilis*	Dai 13080	KJ734260	KJ734264	Zhao et al. (2015)
*Fragiliporia fragilis*	Dai 13559	KJ734261	KJ734265	Zhao et al. (2015)
*Fragiliporia fragilis*	Dai 13561	KJ734262	KJ734266	Zhao et al. (2015)
*Frantisekia mentschulensis*	BRNM 710170	FJ496728	–	Tomšovský et al. (2010)
*Frantisekia mentschulensis*	1377	JN710544	JN710544	Miettinen et al. (2011)
*Ganoderma lingzhi*	Wu 1006-38	JQ781858	–	Zhao et al. (2015)
*Gelatoporia subvermispora*	BRNU 592909	FJ496694	FJ496706	Tomšovský et al. (2010)
*Gloeoporus dichrous*	KHL 11173	EU118627	EU118627	Binder et al. (2005)
*Grammothelopsis subtropica*	Cui 9035	JQ845094	JQ845097	Zhao et al. (2015)
*Heterobasidion annosum*	PFC 5252	KC492906	KC492906	Binder et al. (2013)
*Hornodermoporus martius*	MUCL 41677	FJ411092	FJ393859	Zhao et al. (2015)
*Hypochnicium bombycinum*	MA 15305	FN552537	–	Binder et al. (2013)
*Hypochnicium lyndoniae*	NL 041031	JX124704	JX124704	Binder et al. (2005)
*Junghuhnia crustacea*	X 1127	JN710554	JN710554	Miettinen et al. (2011)
*Junghuhnia crustacea*	X 262	JN710553	JN710553	Miettinen et al. (2011)
*Junghuhnia micropora*	Spirin 2652	JN710559	JN710559	Miettinen et al. (2011)
*Junghuhnia nitida*	KHL 11903	EU118638	EU118638	Binder et al. (2005)
*Loweomyces fractipes*	X 1149	JN710570	JN710570	Miettinen et al. (2011)
*Loweomyces fractipes*	X 1253	JN710569	JN710569	Miettinen et al. (2011)
*Loweomyces fractipes*	X 1250	JN710568	JN710568	Miettinen et al. (2011)
*Mycoacia fuscoatra*	KHL 13275	JN649352	JN649352	Tomšovský et al. (2010)
*Mycoacia nothofagi*	KHL 13750	GU480000	GU480000	Tomšovský et al. (2010)
*Nigroporus vinosus*	X 839	N710576	N710576	Miettinen et al. (2011)
*Nigroporus vinosus*	8182	JN710728	JN710728	Miettinen et al. (2011)
*Obba rivulosa*	KCTC 6892	FJ496693	FJ496710	Miettinen and Rajchenberg (2012)
*Obba valdiviana*	FF 503	HQ659235	HQ659235	Miettinen and Rajchenberg (2012)
*Panus conchatus*	X 1234	JN710579	JN710579	Miettinen et al. (2011)
*Panus strigellus*	INPA 243940	JQ955725	JQ955732	Binder et al. (2013)
*Perenniporia medulla-panis*	MUCL 49581	FJ411088	FJ393876	Robledo et al. (2009)
*Perenniporiella neofulva*	MUCL 45091	FJ411080	FJ393852	Robledo et al. (2009)
*Phlebia unica*	KHL 11786	EU118657	EU118657	Binder et al. (2013)
*Phlebia radiata*	UBCF 19726	HQ604797	HQ604797	Binder et al. (2013)
*Physisporinus sanguinolentus*	BRNM 699576	FJ496671	FJ496725	Tomšovský et al. (2010)
*Physisporinus vitreus*	3163	JN710580	JN710580	Miettinen et al. (2011)
*Piloporia sajanensis*	Mannine 2733a	HQ659239	HQ659239	Miettinen and Rajchenberg (2012)
*Podoscypha venustula*	CBS 65684	JN649367	JN649367	Binder et al. (2013)
*Polyporus tuberaster*	CulTENN 8976	AF516598	AJ488116	Binder et al. (2005)
*Postia guttulata*	KHL 11739	EU11865	EU11865	Kim et al. (2007)
*Pseudolagarobasidium acaciicola*	CBS 115543	DQ517883	–	Miettinen and Rajchenberg (2012)
*Pseudolagarobasidium acaciicola*	CBS 115544	DQ517882	–	Miettinen and Rajchenberg (2012)
*Pseudolagarobasidium belizense*	CFMR 04-31	JQ070173	–	Miettinen and Rajchenberg (2012)
*Skeletocutis amorpha*	Miettinen 11038	FN907913	FN907913	Tomšovský et al. (2010)
*Skeletocutis portcrosensis*	LY 3493	FJ496689	FJ496689	Tomšovský et al. (2010)
*Skeletocutis jelicii*	H 6002113	FJ496690	FJ496727	Tomšovský et al. (2010)
*Skeletocutis novae-zelandiae*	Ryvarden 38641	JN710582	JN710582	Miettinen et al. (2011)
*Spongipellis spumeus*	PRM 891931	HQ728287	HQ729021	Tomšovský et al. (2010)
*Spongipellis spumeus*	BRNM 712630	HQ728288	HQ728288	Tomšovský et al. (2010)
*Spongipellis spumeus*	BRNM 734877	HQ728283	HQ728283	Tomšovský et al. (2010)
*Steccherinum fimbriatum*	KHL 11905	EU118668	EU118668	Tomšovský et al. (2010)
*Steccherinum ochraceum*	Ryberg s.n.	EU118669	EU118670	Larsson (2007)
*Steccherinum ochraceum*	KHL 11902	JQ031130	JQ031130	Miettinen et al. (2011)
*Stereum hirsutum*	NBRC 6520	AB733150	AB733325	Binder et al. (2013)
*Truncospora ochroleuca*	MUCL 39726	FJ411098	FJ393865	Robledo et al. (2009)
*Tyromyces chioneus*	Cui 10225	KF698745	KF698756	Zhao et al. (2015)
*Xanthoporus syringae*	X 339	JN710606	JN710606	Miettinen et al. (2011)
*Xanthoporus syringae*	Cui 2177	DQ789395	–	Miettinen et al. (2011)
*Xanthoporus syringae*	Gothenburg 1488	JN710607	JN710607	Miettinen et al. (2011)
*Elaphroporia ailaoshanens*is	CLZhao 595	MG231568	MG748854	Present study
*Elaphroporia ailaoshanensis*	CLZhao 596	MG231572	MG748855	Present study
*Elaphroporia ailaoshanensis*	CLZhao 597	MG231847	MG748856	Present study
*Elaphroporia ailaoshanensis*	CLZhao 598	MG231823	MG748857	Present study

**Figure 1.**
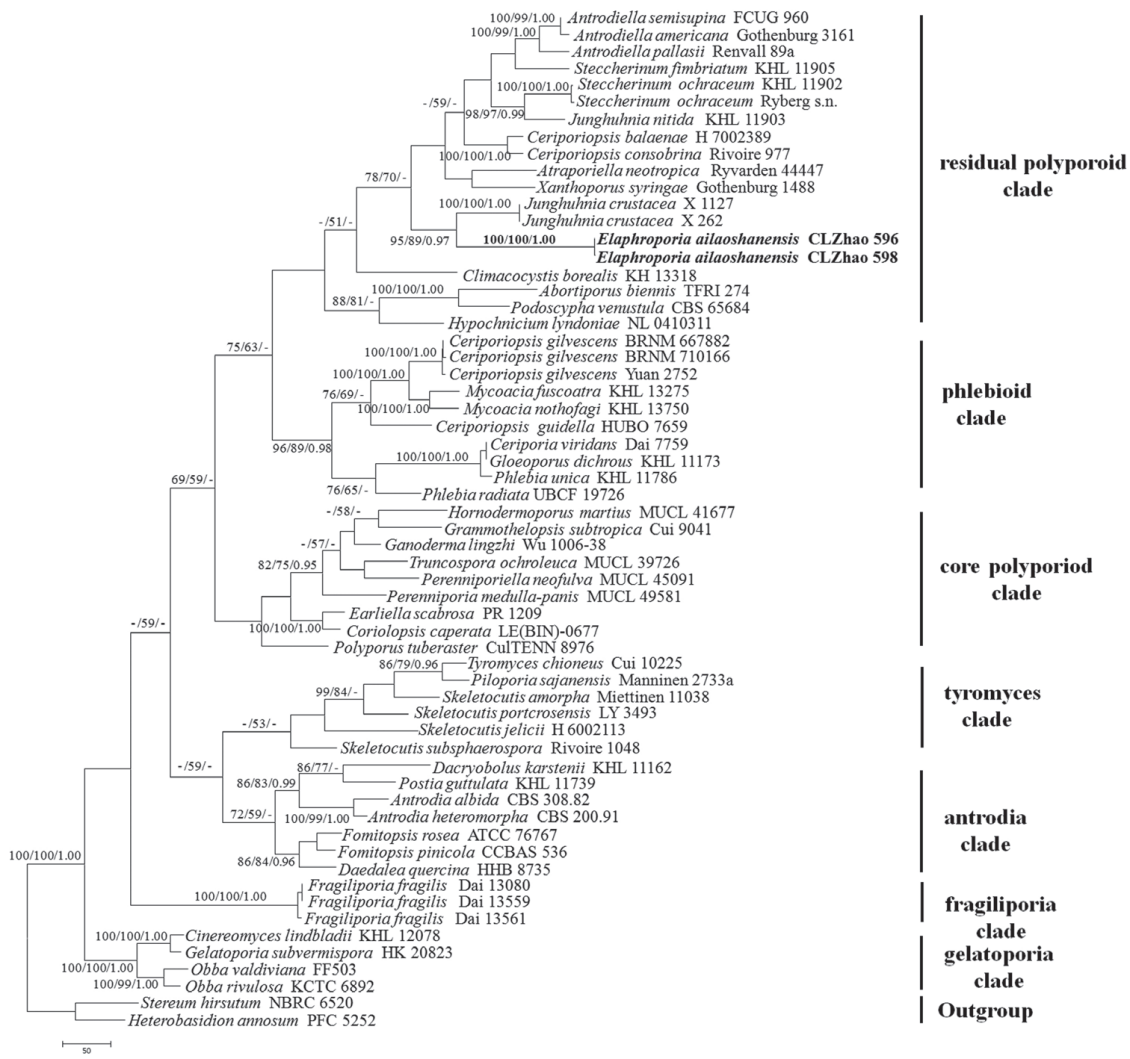
Maximum parsimony strict consensus tree illustrating the phylogeny of *Elaphroporia ailaoshanensis* and related species in Polyporales based on ITS+nLSU sequences. Branches are labelled with parsimony bootstrap values (before slash) higher than 50 % and Bayesian posterior probabilities (after slash) equal to and more than 0.95. Clade names follow Binder et al. (2013).

**Figure 2.**
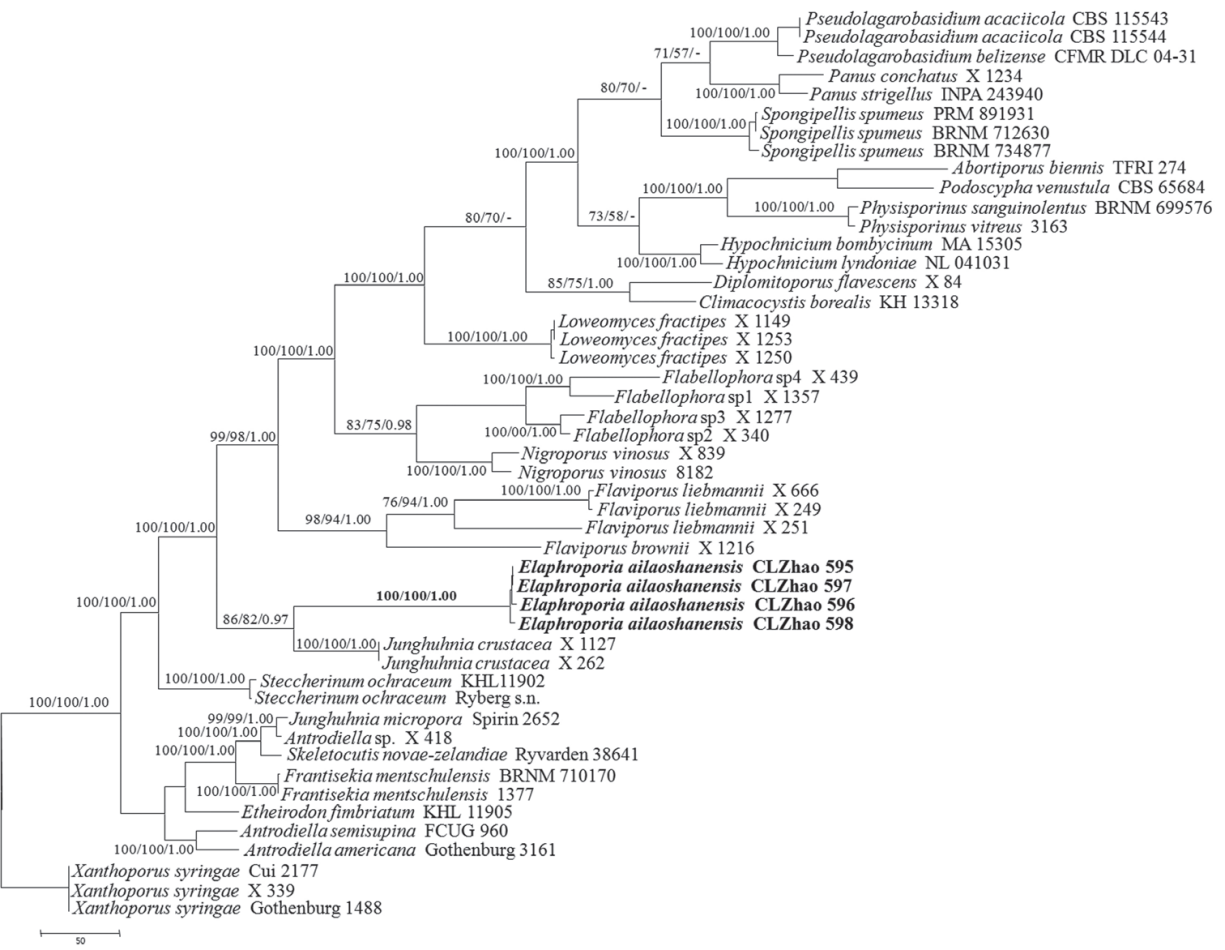
Maximum parsimony strict consensus tree illustrating the phylogeny of *Elaphroporia ailaoshanensis* and related species in the residual polyporoid clade based on ITS+nLSU sequences. Branches are labelled with parsimony bootstrap values (before slash) higher than 50% and Bayesian posterior probabilities (after slash) equal to and more than 0.95. Clade names follow Miettinen et al. (2011).

*Phylogenetic analysis*. Sequencher 4.6 (GeneCodes, Ann Arbor, MI, USA) was used to edit the DNA sequence. Sequences were aligned in MAFFT 6 (Katoh and Toh 2008, http://mafft.cbrc.jp/alignment/server/) using the “G-INS-I” strategy and manually adjusted in BioEdit (Hall 1999). The sequence alignment was deposited in TreeBase (submission ID 21778). Sequences of *Heterobasidion annosum* (Fr.) Bref. and *Stereum hirsutum* (Willd.) Pers. obtained from GenBank were used as outgroups to root trees following Binder et al. (2013) in Figure 1 and *Xanthoporus syringae* (Parmasto) Audet. obtained from GenBank was used as an outgroup to root trees following Miettinen et al. (2011) in the ITS+nLSU analyses (Fig. 2).

Maximum parsimony analysis was applied to the ITS+nLSU dataset sequences. Approaches to phylogenetic analysis followed Li and Cui (2013) and the tree construction procedure was performed in PAUP* version 4.0b10 (Swofford 2002). All characters were equally weighted and gaps were treated as missing data. Trees were inferred using the heuristic search option with TBR branch swapping and 1000 random sequence additions. Max-trees were set to 5000, branches of zero length were collapsed and all parsimonious trees were saved. Clade robustness was assessed using a bootstrap (BT) analysis with 1,000 replicates (Felsenstein 1985). Descriptive tree statistics tree length (TL), consistency index (CI), retention index (RI), rescaled consistency index (RC) and homoplasy index (HI) were calculated for each Maximum Parsimonious Tree (MPT) generated. Sequences were also analysed using Maximum Likelihood (ML) with RAxML-HPC2 through the Cipres Science Gateway (www.phylo.org; Miller et al. 2009). Branch support for ML analysis was determined by 1000 bootstrap replicates.

MrModeltest 2.3 (Posada and Crandall 1998, Nylander 2004) was used to determine the best-fit evolution model for each data set for Bayesian Inference (BI). Bayesian Inference was calculated with MrBayes 3.1.2 with a general time reversible (GTR) model of DNA substitution and a gamma distribution rate variation across sites (Ronquist and Huelsenbeck 2003). Four Markov chains were run for 2 runs from random starting trees for 5 million generations (Fig. 1), for 3 million generations (Fig. 2) and trees were sampled every 100 generations. The first one-fourth generations were discarded as burn-in. A majority rule consensus tree of all remaining trees was calculated. Branches that received bootstrap support for maximum likelihood (BS), maximum parsimony (BP) and Bayesian posterior probabilities (BPP) greater than or equal to 75 % (BP) and 0.95 (BPP) respectively, were considered as significantly supported.

### Phylogeny results

The ITS+nLSU dataset (Fig. 1) included sequences from 60 fungal specimens representing 52 taxa. The dataset had an aligned length of 2143 characters, of which 1251 characters were constant, 206 parsimony-uninformative and 686 parsimony-informative. MP analysis yielded 6 equally parsimonious trees (TL = 4744, CI = 0.322, HI = 0.678, RI = 0.578, RC = 0.186). The best-fit model for ITS+nLSU alignment estimated and applied in the BI was GTR+I+G, lset nst = 6, rates = invgamma; prset statefreqpr = dirichlet (1,1,1,1). BI resulted in a similar topology with an average standard deviation of split frequencies = 0.001755.

The phylogenetic tree (Fig. 1), inferred from ITS+nLSU sequences, demonstrated seven major clades for 60 sampled species of the Polyporales. The new genus *Elaphroporia* fell into the Meruliaceae within the residual polyporoid clade. It was closely related to *Junghuhnia crustacea* (Jungh.) Ryvarden with a good support (95% BS, 89% BP, 0.97 BPP).

The ITS+nLSU (Fig. 2) dataset included sequences from 48 fungal specimens representing 31 taxa. The dataset had an aligned length of 2163 characters, of which 1429 characters were constant, 169 parsimony-uninformative and 565 parsimony-informative. MP analysis yielded 8 equally parsimonious trees (TL = 2806, CI = 0.423, HI = 0.576, RI = 0.673, RC = 0.285). The best-fit model for ITS+nLSU alignment estimated and applied in the BI was GTR+I+G, lset nst = 6, rates = invgamma; prset statefreqpr = dirichlet (1,1,1,1). BI resulted in a similar topology with an average standard deviation of split frequencies equal to 0.005758.

A further phylogeny (Fig. 2) inferred from the combined ITS+nLSU sequences was obtained for 48 fungal specimens representing 31 taxa within the residual polyporoid clade and demonstrated that the new genus formed a monophyletic entity with a high 100 % BS, 100 % BP and 1.00 BPP and sisters to *Junghuhnia crustacea* and then grouped with *Flaviporus* and *Steccherinum*.

## Taxonomy

### Elaphroporia

Taxon classificationFungiPolyporalesMeruliaceae

Z.Q. Wu & C.L. Zhao gen. nov.

823915

#### Diagnosis.

Differs from other genera in Polyporales by resupinate basidiocarps becoming rigid and light-weight upon drying, a monomitic hyphal system, thick-walled generative hyphae bearing both clamp connections and simple septa and hyaline, thin-walled, smooth, IKI–, CB– basidiospores.

#### Type species.

*Elaphroporia ailaoshanensis* Z.Q. Wu & C.L. Zhao.

#### Etymology.

*Elaphroporia* (Lat.): referring to the basidiocarps light-weight upon drying.

Basidiocarps annual, resupinate, becoming rigid and light-weight up on drying. Pore surface cream to pale yellow when fresh, turning to yellow upon drying. Hyphal system monomitic; generative hyphae thick-walled bearing both clamp connections and simple septa, slightly amyloid, CB+. Basidiospores ellipsoid, hyaline, thin-walled, smooth, IKI–, CB–.

### Elaphroporia ailaoshanensis

Taxon classificationFungiPolyporalesMeruliaceae

Z.Q. Wu & C.L. Zhao sp. nov.

823916

Figs 3 , 4

#### Diagnosis.

This species is distinguished by the cream to yellow pore surface upon drying; pores angular, 7–9 per mm. Hyphal system monomitic; generative hyphae thick-walled bearing both clamp connections and simple septa, slightly amyloid, CB+. Basidiospores ellipsoid, hyaline, thin-walled, smooth, IKI–, CB–, 1.9–2.5 × 1.5–2 µm.

**Figure 3.**
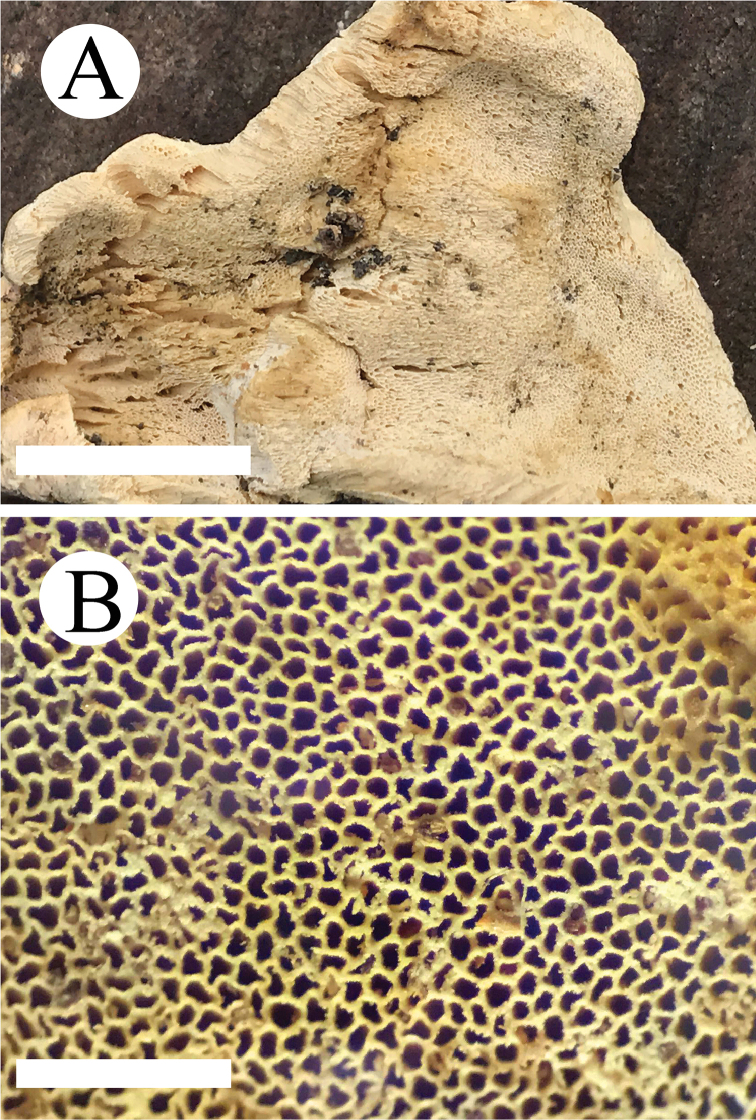
Basidiomata of *Elaphroporia ailaoshanensis* (holotype). Scale bars: 1 cm (**A**); 1 mm (**B**).

**Figure 4.**
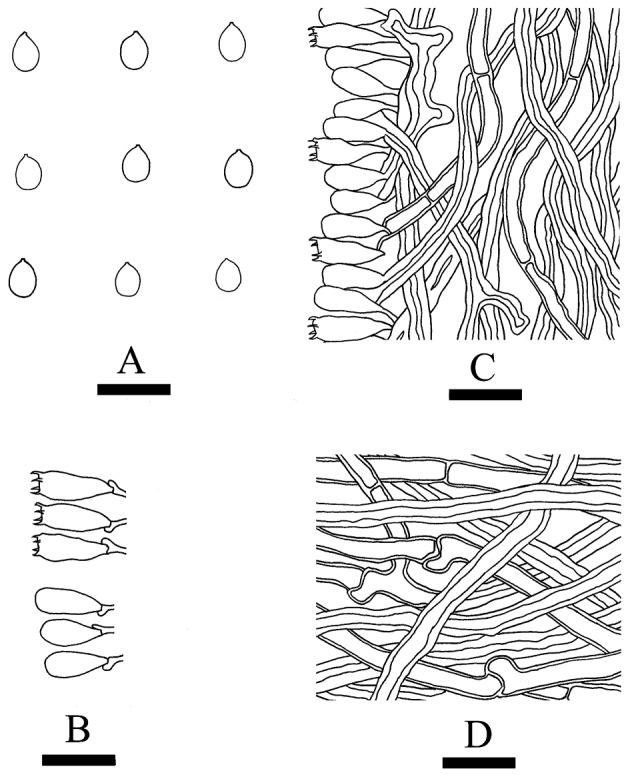
Microscopic structures of *Elaphroporia ailaoshanensis* (drawn from the holotype). **A** Basidiospores **B** Basidia and basidioles **C** Hyphae from trama **D** Hyphae from subiculum.

#### Holotype

CHINA. Yunnan Province: Jingdong county, Ailaoshan Nature Reserve, 2 October 2016, on the angiosperm trunk, CLZhao 595 (Holotype in SWFC).

#### Etymology.

*Ailaoshanensis* (Lat.): referring to the locality (Ailaoshan) of the type specimens.

#### Basidiocarps.

Annual, resupinate, easy to separate from substrate, soft corky when fresh, without odour or taste when fresh, becoming rigid and light-weight up on drying, up to 5 cm long, 3.5 cm wide, 4 mm thick at centre. Pore surface cream to pale yellow when fresh, turning to yellow upon drying; pores angular, 7–9 per mm; dissepiments thin, entire. Sterile margin narrow, cream, up to 1 mm wide. Subiculum thin, cream, corky, up to 0.2 mm thick. Tubes concolorous with pore surface, hard corky, up to 3.8 mm long.

#### Hyphal structure.

Hyphal system monomitic; generative hyphae thick-walled, slightly amyloid, CB+; tissues unchanged in KOH.

#### Subiculum.

Generative hyphae hyaline, thick-walled bearing both clamp connections and simple septa, simple septa more frequent than clamps, occasionally branched, interwoven, 3.5–5.5 µm in diam.

#### Tubes.

Generative hyphae hyaline, thick-walled bearing simple septa only, occasionally branched, 3–5 µm in diameter. Cystidia and cystidioles absent; basidia clavate, with four sterigmata and a basal clamp connection, 10.5–14.5 × 3.5–4.5 µm; basidioles dominant, in shape similar to basidia, but slightly smaller.

#### Spores.

Basidiospores ellipsoid, hyaline, thin-walled, smooth, IKI–, CB–, (1.7–)1.9–2.5(–2.9) × (1.3–)1.5–2(–2.2) µm, L = 2.29 µm, W = 1.74 µm, Q = 1.33–1.81 (n = 120/4).

#### Additional specimens examined

**(paratypes)**. CHINA. Yunnan Province: Jingdong county, Ailaoshan Nature Reserve, 2 October 2016, on the angiosperm trunk, CLZhao 596, CLZhao 597, CLZhao 598 (SWFC).

## Discussion

In the present study, a new genus, *Elaphroporia*, is described based on phylogenetic analyses and morphological characters. The genus has unique morphological characters in Meruliaceae.

Previously, seven clades were found in the Polyporales: antrodia clade, core polyporoid clade, fragiliporia clade, gelatoporia clade, phlebioid clade, residual polyporoid clade and tyromyces clade (Binder et al. 2013, Zhao et al. 2015). According to these results based on the combined ITS+nLSU sequence data (Fig. 1), the new genus is nested into the residual polyporoid clade with strong support (100 % BS, 100 % BP, 1.00 BPP).

Miettinen et al. (2011) analysed a higher-level phylogenetic classification of the residual polyporoid clade morphological plasticity in a group of the polypores, and showed that the natural genera could mostly be characterised morphologically and poroid and hydnoid species belong to separate genera. The current phylogeny shows that the genus *Elaphroporia* falls into the residual polyporoid clade and belongs to the family Meruliaceae (Figs 1, 2). Furthermore, the new genus is closely related to *Junghuhnia* and then grouped with *Flaviporus* and *Steccherinum* based on ITS+LSU-nrRNA gene regions with a strong support (100 % BS, 100 % BP, 1.00 BPP; Fig. 1). However, morphologically *Junghuhnia* differs from *Elaphroporia* by a dimitic hyphal system and presence of cystidia (Núñez and Ryvarden 2001, Ryvarden and Melo 2014). *Flaviporus* is separated from *Elaphroporia* by the dark brown to bay pileus, a dimitic hyphal system and presence of the metuloid cystidia (Murrill 1905). *Steccherinum* differs in its odontioid to hydnoid hymenophore and cyanophilous basidiospores (Bernicchia and Gorjón 2010).

Morphologically, *Elaphroporia* resembles *Ceriporia* Donk and *Phlebiporia* Jia J. Chen, B.K. Cui & Y.C. Dai. *Ceriporia* is similar to *Elaphroporia* in an annual growth habit with poroid hymenophore, a monomitic hyphal structure and hyaline, thin-walled and smooth basidiospores. In addition, both genera cause a white rot. However, *Ceriporia* differs from *Elaphroporia* by the generative hyphae IKI–, CB– (Jia et al. 2014). Additionally, in molecular studies, *Ceriporia* fell into the phlebia clade (Miettinen and Larsson 2011, Miettinen and Rajchenberg 2012, Miettinen et al. 2011, Binder et al. 2013) which is also the same as in the authors’ study (Fig. 1). *Phlebiporia* is similar to *Mellipora* by having the poroid hymenophore and the generative hyphae bearing both simple septa and clamp connections, but it is separated from *Elaphroporia* by having dextrinoid generative hyphae, tissues becoming brownish in KOH and presence of thin-walled quasi-binding hyphae in the subiculum (Chen and Cui 2014).

Polypores are an extensively studied group of Basidiomycota (Gilbertson and Ryvarden 1987, Núñez and Ryvarden 2001, Dai 2012, Ryvarden and Melo 2014), but the Chinese polypore diversity is still not well known, especially in subtropics and tropics, from where many recently described taxa of polypores were discovered (Song et al. 2014, 2016, Zhou et al. 2015, 2016, Nie et al. 2017, Yuan et al. 2017). The new genus in the present study, *Elaphroporia*, is also from the subtropics. It is possible that new polypore taxa will be found after further investigations and molecular analyses.

## Supplementary Material

XML Treatment for Elaphroporia

XML Treatment for Elaphroporia ailaoshanensis
